# A Concise Synthesis of Glycolipids Based on Aspartic Acid Building Blocks

**DOI:** 10.3390/molecules171011346

**Published:** 2012-09-25

**Authors:** Trinidad Velasco-Torrijos, Lorna Abbey, Roisin O’Flaherty

**Affiliations:** Department of Chemistry, National University of Ireland Maynooth, Co. Kildare, Ireland

**Keywords:** synthetic glycolipids, glycosylated amino acids, glycomimetics, aspartic acid, racemization

## Abstract

L-Aspartic acid building blocks bearing galactosyl moieties were used to synthesise glycolipid mimetics of variable hydrocarbon chain length. The glycolipids were readily prepared through amide bond formation using the TBTU/HOBt coupling methodology. It was observed that, under these conditions, activation of the α-carboxylic acid of the intermediates led to near complete racemisation of the chiral centre if the reaction was carried out in the presence of a base such as triethylamine. The enantiomerically pure glycolipids were obtained after careful consideration of the synthetic sequence and by performing the coupling reactions in the absence of base.

## 1. Introduction

**Figure 1:**
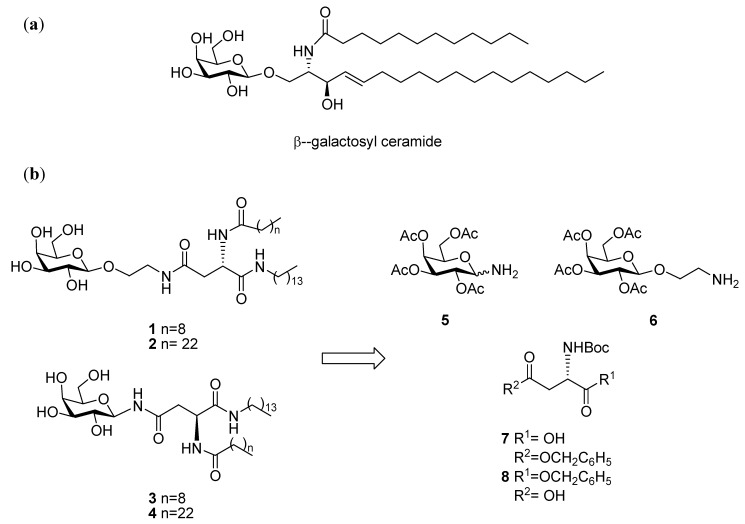
(**a**) Structure of the naturally occurring glycolipid β-galactosyl ceramide. (**b**) Structure of the galactosyl amines **5** [17,18] and **6** [19] and of the commercially available L-aspartic acid derivatives **7** and **8**, used for the modular synthesis of glycolipid mimetics **1**–**4**.

Synthetic glycomimetics have been the subject of much research activity in the field of carbohydrate chemistry. The important role of carbohydrates in biological systems has prompted the development of different types of glycomimetics intended for diverse applications, such as therapeutic leads [1,2], novel materials [3], biosensors and diagnostic tools [4,5]. Glycolipid mimetics [6], in particular synthetic derivatives of biologically relevant ceramides (such as galactosyl ceramides, shown in Figure 1a), have attracted the attention of many carbohydrate chemists over recent years [7].

Amino acids that allow for side chain functionalization with glycosyl moieties, such as serine and aspartic acid, have been popular choices as the starting point for the preparation of glycolipid analogues [8,9]. The carboxylic acid present on the aspartic acid side chain offers the possibility for attachment of mono or oligosaccharides, while both the amino and carboxylic acid groups at the α-carbon allow for further functionalization. Due to the biological relevance of *N*-linked glycosides, this type of building blocks has been used predominantly in the synthesis of glycopeptides and glycopeptoids and hence, numerous examples of such compounds can be found in the literature [10,11,12,13]. In this study we report our investigations towards the synthesis of galactosylated building blocks based on: (i) orthogonally protected; (ii) enantiomerically pure and (iii) commercially available L-aspartic acid derivatives, as we intend to expand their application to the preparation of glycolipid mimetics. These non-natural glycolipids may be bioactive as neuroprotective agents [14] and/or may be used in materials or formulation science [15]. The nature of the building blocks should allow for a modular approach which could lead to the facile preparation of a small collection of glycolipids of different fatty acids chain lengths, such as **1**–**4**, shown in Figure 1b. This feature of the glycolipid structure affects strongly its physicochemical characteristics, as well as its potential biological activity [16].

We have initially focused our attention on derivatives of decanoic acid (C-10), such as **1** and **3**, and tetracosanoic acid (C-24), such as **2** and **4**, as representative examples of medium and long fatty acid chain lengths. In glycolipids **1** and **2**, the galactosyl moiety is connected to the aspartic acid by a flexible ethylene-type linker, while glycomimetics **3** and **4** resemble the native *N*-linked glycosides, as the acid conjugation occurs directly at the anomeric center. It is therefore expected that both sets of compounds would have different degrees of conformational freedom, which in turn may have an effect on their potential biological activities and physical properties.

## 2. Results and Discussion

### Synthesis of the Glycolipids

**Scheme 1:**
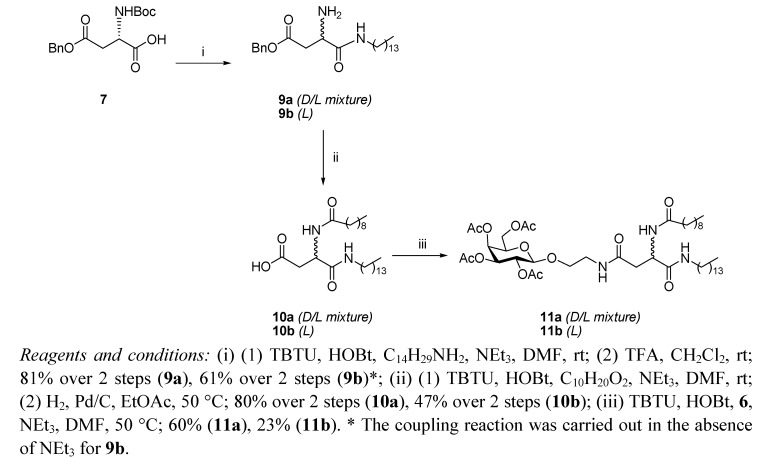
Synthesis of glycolipids **11a** and **11b**.

Both the galactosyl amines **5** [17,18] and **6** [19] used in the syntheses described herein are readily prepared from D-galactose pentacetate following procedures described in literature. Our initial approach to the glycolipid mimetics **1**–**4** involved a convergent synthesis (Scheme 1), whereby the *N*-Boc-γ-benzyl ester protected L-aspartic acid **7** was coupled to tetradecylamine using standard TBTU/HOBt activation conditions in the presence of triethylamine. Subsequent removal of the *N*-Boc protecting group with TFA afforded the amine **9a**, which was acylated with decanoic acid using the above mentioned TBTU/HOBt methodology. Hydrogenolysis of the side chain benzyl ester was carried out at 50 °C to enhance solubility and it afforded carboxylic acid **10a**, which was then coupled to the primary amine of galactosyl derivative **6**, to yield the acetyl protected glycolipid **11a**. The ^1^H-NMR spectrum of **11a** showed distinct duplication of every expected signal in a 1:1 ratio. To rule out possible conformational exchange equilibrium, variable temperature ^1^H-NMR spectra of compound **11a** were recorded in *d*_6_-DMSO. No coalescence of the signals was observed at temperatures as high as 80 °C, which confirmed that glycolipid **11a** was, in fact, a mixture of diastereoisomers. 

The unexpected racemisation of the chiral α-carbon of the L-aspartic acid derivative **7** takes place in the first step of the synthesis. Although the use of TBTU and HOBt as coupling reagents is a very standard procedure in peptide synthesis [20], the activation of the α-carboxylic acid under these conditions is likely to increase the acidity of the α-proton in **7** and it may be abstracted in the presence of a base such as triethylamine. This is further supported by the disappearance of the optical activity of compound **9a** [[α]^22^_D_ = 0 (c 1.55, CHCl_3_)], while if the same coupling reaction is carried out in the absence of triethylamine, a specific optical rotation value is obtained for the L-enantiomer, compound **9b** [[α]^22^_D_ = +2.5 (c 1.55, CHCl_3_)]. The effects on reaction yields and racemisation of the products, caused by different bases and activating reagents commonly used in peptide couplings, have been extensively reviewed in the literature [21]. Most of the published procedures reporting amide bond formation of *N*-Boc aspartic acid **7** involve the use of carbodiimide-type coupling reagents [22], formation of activated esters, such as pentafluorophenyl derivatives [23], or mixed anhydrides [24]. However, no compromise of the optical purity of the resulting aspartate derivatives when using uronium-type reagents (such as TBTU or HBTU) has been explicitly reported so far, to the best of our knowledge [25,26]. The mixture of D and L diastereoisomers of glycolipid **11a** could not be separated by flash column chromatography or by recrystallization.

**Scheme 2:**
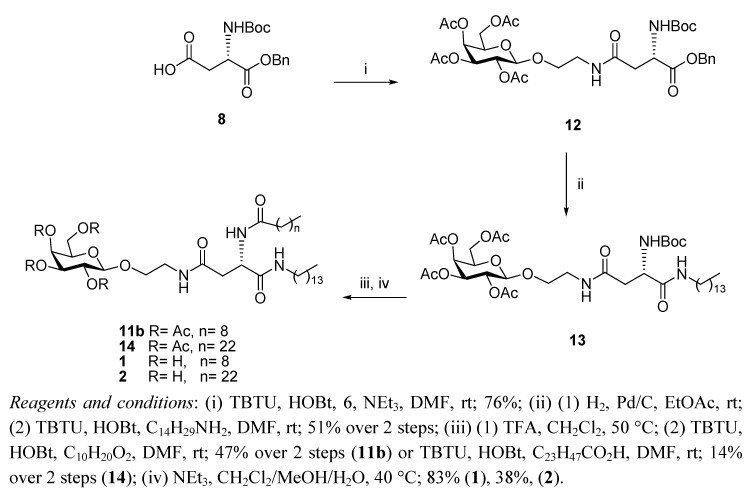
Synthesis of *O*-glycolipids **1** and **2**.

The same synthetic sequence as described above was carried out on the L-enantiomer **9b**. Although this route allowed access to sufficient amounts of diastereomerically pure **11b**, we decided to investigate a different synthetic sequence that may result in an overall higher yield for the enantiomerically pure glycolipids, as outlined in Scheme 2. In the first step of the reviewed scheme, the free amino galactosyl derivative **6** was coupled to the *N*-Boc aspartic acid benzyl ester **8**, which bears the free carboxylic acid at the side chain, to give the orthogonally protected compound **12**. The benzyl ester on **12** was removed by hydrogenolysis and the resulting carboxylic acid at the α-carbon was then carefully reacted again with the TBTU/HOBt system, followed by the addition of tetradecylamine. To avoid racemisation of the chiral carbon in this crucial step, this reaction was carried out in the absence of base. Under these conditions, enantiomerically pure **13** was successfully obtained, albeit in a moderate yield (51% over two steps). This building block was then reacted with TFA to cleave the *N*-Boc group and the corresponding amine was acylated with pre-activated decanoic acid (stirred with TBTU/HOBt prior to addition) to lead to the protected glycolipid **11b**. Acylation of the amine derived from **13** by treatment with TBTU/HOBt and tetracosanoic acid instead gave the longer C-24 compound **14**.The hydrolysis of the acetyl protecting groups on the galactosyl moiety of both derivatives **11a** and **14** was initially attempted following standard procedures, such as the Zemplén deprotection or reaction with hydrazine [27]. However, these conditions proved to be rather harsh, resulting in amide bond hydrolysis and degradation of the glycolipids. Enzyme catalysed acetolyisis was also considered, using both immobilized enzymes (such as CALB, *Candida antarctica *lipase [28], immobilized as Novazym 435) and soluble lipases (such as CRL, *Candida rugosa *lipase) [29]. These and many other lipases have been reported to chemoselectively achieve total or partial deacetylation of protected glycosides. However, the success of enzyme-catalysed reactions is often highly dependent on substrate structure, and we found that, perhaps due to the steric bulk imposed by the hydrocarbon chains, not even partial deacetylation of any of the glycolipids could be achieved. The deprotection of **11b** and **14** was most successfully carried out with mild base catalysis in a heterogenous mixture of triethylamine and dichloromethane/methanol/water at 40 °C, to give the corresponding glycolipids **1** and **2**.

**Scheme 3:**
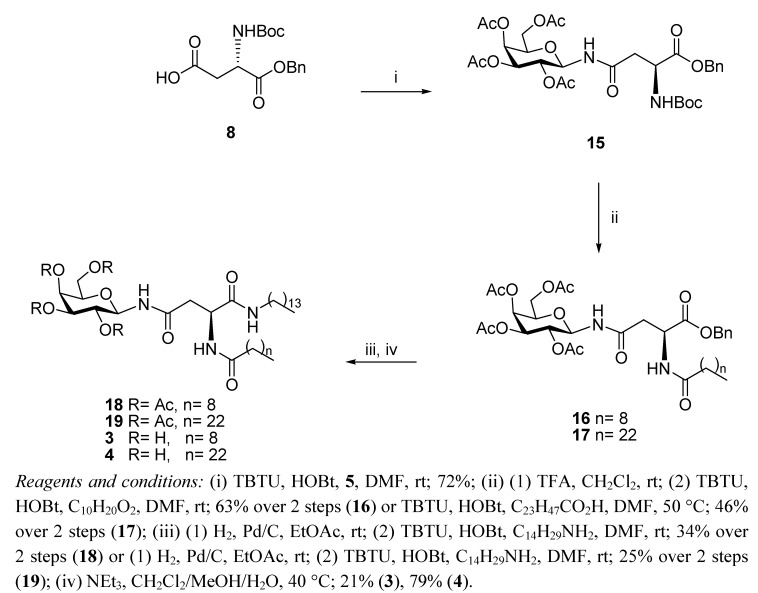
Synthesis of *N*-glycolipids **3** and **4**.

Similar considerations regarding the preservation of the chirality of the L-aspartic acid asymmetric α-carbon were observed in the reviewed syntheses of the anomeric *N*-linked glycolipid analogues **3** and **4** (Scheme 3). In this case, the synthesis starts again with the direct coupling of galactosyl amine **5 **with the *N*-Boc aspartic acid benzyl ester **8** to give building block **16** [30]. In order to avoid side reactions due to the increased acidity of the resulting anomeric amide, the reaction was carried out with TBTU/HOBt but in the absence of an added base. The next step involved the removal of the *N*-Boc group of **15** with TFA, and the corresponding amine was acylated with either pre-activated decanoic or tetracosanoic acid, to give the corresponding intermediates **16** and **17**, respectively. It was expected that the presence of the long hydrocarbon chains would introduce steric hindrance and minimize the risk of intramolecular cyclization to yield aspartimide-type by-products when attempting the coupling of the α-carboxylic acid, as this is a well known side reaction in glycopeptides and glycoprotein synthesis [31,32]. Indeed, after **16** and **17** underwent hydrogenolysis, the corresponding carboxylic acids were subjected to reaction with tetradecylamine mediated by TBTU/HOBt to give the enantiomerically pure glycolipids **18** and **19**, and no significant formation of cyclic products could be observed. Since Zemplén deacetylation may have involved too harsh conditions for the final deprotection of the glycolipids **11b** and **14** described earlier, we used again the mildly basic hydrolysis method described above to access derivatives **3** and **4**. It must be noted that the solubility of the C-24 tetracosanoic acid derivatives, **2** and **4** is very poor (both in water and in most common solvents), when compared to that of the C-10 decanoic glycolipids **1** and **3**. This is likely to hamper potential applications of the longer chain analogues.

## 3. Experimental

### General Methods

All chemicals purchased were reagent grade and used without further purification unless stated otherwise. Dichloromethane was freshly distilled over CaH_2_ prior use. Anhydrous dimethylformamide (DMF) was purchased from Sigma Aldrich. Molecular sieves (MS) used for glycosylation and coupling reactions were 8–12 mesh and were flame dried prior to use. Reactions were monitored with thin layer chromatography (TLC) on Merck Silica Gel F_254_ plates, using mixtures of hexane/ethyl acetate unless otherwise stated. Detection was effected either by visualisation in UV light and/or charring in a mixture of 5% sulphuric acid-EtOH or phosphomolybdic acid-EtOH. NMR spectra were obtained on a Bruker Avance 300 spectrometer. Proton and carbon signals were assigned with the aid of 2D-NMR experiments and DEPT experiments for novel compounds. The 2D-NMR experiments included COSY and HCCOSW, which is an HSQC type of experiment. Better resolution of the signals was observed when using the HCCOSW experiments than with conventional HSQC experiments. Chemicals shifts for ^1^H-NMR are reported in ppm relative to residual solvent proton. Flash chromatography was performed with Merck Silica Gel 60, using adjusted mixtures of hexane/ethyl acetate unless otherwise stated. Optical rotations were obtained using an AA-100 polarimeter. [α]^25^ values are given in 10^−1^ cm^2^·g^−1^. The melting points were obtained using a Stuart Scientific SMP1 melting point apparatus and are uncorrected. High resolution mass spectrometry (HRMS) were performed on an Agilent-LC 1200 Series coupled to a 6210 Agilent Time-Of-Flight (TOF) mass spectrometer equipped with an electrospray source both positive and negative (ESI+/−) or in a MALDI-QTOF Premier MS SYSTEM, using an α-cyano-4-hydroxy cinnamic acid matrix. Infrared spectra were obtained as a film on NaCl plates in the region 4000–400 cm^−1^ on a Nicolet Impact 400D spectrophotometer.

*N^4^-[2-O-(2,3,4,6-Tetra-O-acetyl-β-D-galactopyranosyl)-ethyl]-N^2^-tert-butoxycarbonyl-L-asparagine benzyl ester *(**12**). HOBt (0.09 g, 0.68 mmol), followed by NEt_3_ (0.18 mL, 1.23 mmol), were added to a stirring solution of *N*-Boc-L-Asp-OBn **8** (0.2 g, 0.61 mmol) and TBTU (0.22 g, 0.6 mmol) dissolved in anhydrous DMF (10 mL), under N_2_ at rt. It was stirred for 30 min and **6** (0.29 g, 0.74 mmol) dissolved in anhydrous DMF (1.2 mL) was added dropwise. It was stirred for 18 h. The reaction mixture was concentrated under reduced pressure, diluted with ethyl acetate and washed succesively with HCl 0.1 N, aqueous sat. NaHCO_3_ solution, and brine. Flash chromotagraphy (hexane:ethyl acetate, 1:1) afforded **12** as a white solid (0.33 g, 76%). [α]^22^_D_ +6.9 (c 1.35, CH_2_Cl_2_,); IR (NaCl film): 3374.7, 2978.0, 1750.7, 1665.8, 1499.3, 1368.8, 1224.3, 1167.9, 1124.3,1057.2 cm^−1^; ^1^H-NMR (300 MHz, CDCl_3_): δ 7.34 (bs, 5 H, *H*-Ph), 6.01 (t,*J* = 5.1 Hz, 1 H, CH_2_CH_2_N*H*CO), 5.76 (d, *J =* 8.1 Hz, 1 H, N*H*COC(CH_3_)_3_), 5.39 (dd, *J =*0.6 Hz, *J =* 3.3 Hz, 1 H, *H*-4), 5.20–5.16 (m, 3 H, overlap of *H*-2, C*H*_2_Ph), 5.02 (dd, *J =* 3.3 Hz, *J =* 10.2 Hz, 1 H, *H*-3), 4.57–4.54 (m, 1 H, H-α), 4.44 (d, *J =* 7.8 Hz, 1 H, *H*-1), 4.18–4.13 (m, 2 H, overlap of *H*-6, *H*-6'), 3.93–3.89 (m, 1 H, *H*-5), 3.86–3.80 (m, 1 H, 1 H of OC*H*_2_CH*_2_*NH), 3.66–3.59 (m, 1 H, 1 H of OC*H*_2_CH*_2_*NH), 3.46–3.38 (m, 2 H, OCH_2_C*H_2_*NH), 2.91 (dd, *J =* 5.7 Hz, *J =* 17.4 Hz, 1 H, *H*-β), 2.71 (dd, *J =* 4.5 Hz, *J =* 15.9 Hz, 1 H, H-β'), 2.15 (s, 3 H, O(CO)C*H*_3_), 2.05 (s, 6 H, O(CO)C*H*_3_ × 2), 1.99 (s, 3 H, O(CO)C*H*_3_), 1.42 (s, 9 H, COC(C*H*_3_)_3_); ^13^C-NMR (75 Hz, CDCl_3_): δ 171.38, 170.37, 169.76 (each *C*O), 155.55 (*C*-Ph), 128.51, 128.09 (*C*H-Ph), 101.42 (*C*-1), 79.01 (CO*C*(*C*H_3_)_3_), 70.89 (*C*-5), 70.68 (*C*-2), 68.91 (*C*-3), 67.25 (*C*H_2_Ph), 66.96 (*C*-4), 61.35 (*C*-6), 50.47 (*C*-α), 39.18, 37.72 (each O*C*H_2_*C*H*_2_*NH), 37.12 (*C*-β), 28.29 (COC(*C*H_3_)_3_), 20.82, 20.57 (overlap of O(CO)*C*H_3_); HRMS (MS-TOF): [M+H]^+^ calcd. for C_32_H_44_N_2_O_15_: 697.2181 found 697.2800.

*N^4^-[2-O-(2,3,4,6-Tetra-O-acetyl-β-D-galactopyranosyl)-ethyl]-N^2^-tert-butoxycarbonyl-L-asparagine tetradecylamide *(**13**). To a solution of **12** (0.120 g, 0.17 mmol) in ethyl acetate (6 mL), Pd/C 10% w/w (0.012 g, 10% w/w) was added. The resulting slurry was stirred under H_2_ gas for 4 h. The mixture was then filtered through a Celite cake and the filtrate was concentrated under vacuum to afford the corresponding carboxylic acid as an off-white solid, which was used without further purification (0.094 g, 90%). ^1^H-NMR (300 MHz, CDCl_3_): δ 6.53 (bs, 1 H, CH_2_CH_2_N*H*CO), 5.86 (d, *J* = 5.4 Hz, 1 H, N*H*COC(CH_3_)_3_), 5.41 (d, *J *= 3.3 Hz, 1 H, *H*-4), 5.17 (dd, *J* = 7.8 Hz, *J* = 10.2 Hz, 1 H, *H*-2), 5.00 (dd, *J* = 3.3 Hz, *J* = 13.8 Hz, 1 H, *H*-3), 4.49 (d, *J* = 7.8 Hz, 1 H, *H*-1), 4.45–4.42 (m, 1 H, H-α), 4.20–4.11 (m, 2 H, overlap of *H*-6, *H*-6'), 3.96–3.92 (m, 1 H, *H*-5) 3.90–3.85 (m,1 H, 1 H of OC*H*_2_CH*_2_*NH), 3.75–3.68 (m, 1 H, 1 H of OC*H*_2_CH*_2_*NH), 3.54–3.39 (m, 2 H, OCH_2_C*H_2_*NH), 2.91 (d, *J* = 15.6 Hz, 1 H, H-β), 2.72 (dd, *J* = 8.49 Hz, *J* = 15.9 Hz, 1 H, H-β'), 2.17, 2.13, 2.05, 1.98 (each s, 3 H, O(CO)C*H*_3_), 1.42 (s, 9 H, COC(C*H*_3_)_3_); HRMS (MS-TOF): [M+K]^+^ calcd. for C_25_H_38_N_2_O_15_: 645.1904 found 645.1899. HOBt (0.034 g, 0.25 mmol) was added to a stirring solution of the carboxylic acid obtained as described above (0.140 g, 0.23 mmol), tetradecylamine (0.06 g, 0.28 mmol), and TBTU (0.081 g, 0.25 mmol) dissolved in anhydrous DMF, (12 mL) at rt. It was stirred for 18 h. The reaction mixture was concentrated *in vacuo*, diluted with ethyl acetate and washed with brine. Flash chromotagraphy (ethyl acetate) afforded **13** as a white solid (0.120 g, 56%). [α]^22^_D_ = +8.8 (c 0.75, CH_2_Cl_2_,); IR (NaCl film): 3316.3, 3091.3 2919.9, 2851.3, 1748.0, 1687.1, 1646.1, 1548.9, 1524.1, 1467.4, 1434.6, 1369.2, 1368.8, 1230.2, 1171.0, 1055.3 cm^−1^; ^1^H-NMR (300 MHz, CDCl_3_): δ 6.87 (bs, 1 H, CON*H*C_14_CH_29_), 6.23 (t, *J* = 10.5 Hz, 1 H, CH_2_CH_2_N*H*CO), 6.14 (d, *J* = 8.1 Hz, 1 H, N*H*COC(CH_3_)_3_), 5.38 (d, *J* = 2.7 Hz, 1 H, *H*-4), 5.15 (dd, *J* = 7.8 Hz, *J* = 10.5 Hz, 1 H, *H*-2), 5.01 (dd, *J* = 3.3 Hz, *J* = 10.5 Hz, 1 H, *H*-3), 4.49 (d, *J* = 7.8 Hz, 1 H, *H*-1), 4.40–4.39 (m, 1 H, *H*-α), 4.15–4.11 (m, 2 H, overlap of *H*-6, *H*-6'), 3.94–3.87 (m, 1 H, *H*-5), 3.86–3.81 (m, 1 H, 1 H of OC*H*_2_CH*_2_*NH), 3.67–3.60 (m, 1 H, 1 H of OC*H*_2_CH*_2_*NH), 3.49–3.34 (m, 2 H, OCH_2_C*H_2_*NH), 3.22–3.15 (m, 2 H, NHC*H*_2_C_13_H_27_), 2.71 (dd, *J* = 4.2 Hz, *J* = 15.6 Hz, 1 H, H-β), 2.51 (dd, *J* = 6.6 Hz, *J* = 15.6 Hz, 1 H, H-β'), 2.14, 2.07, 2.02, 1.96 (each s, 3 H, O(CO)C*H*_3_), 1.42 (m, 11 H, overlap of COC(C*H*_3_)_3_, NHCH_2_C*H*_2_C_12_H_25_), 1.23 (bs, 22 H, NHC_2_H_4_(C*H*_2_)_11_CH_3_), 0.87–0.83 (t, *J* = 6.6 Hz, 3 H, NHC_13_H_26_C*H*_3_); ^13^C-NMR (75 Hz, CDCl_3_): δ 171.17, 170.88, 170.35, 170.18, 170.05, 169.85, 155.73 (*C*O), 101.31 (*C*-1), 80.14 (CO*C*(CH_3_)_3_), 70.81, 70.72 (*C*-5, *C*-3), 68.91. (*C*-2), 68.56 (NH*C*H_2_C_13_H_27_NH), 66.98 (*C*-4), 61.33 (*C*-6), 51.08 (*C*-α), 39.61, 39.22 (each O*C*H_2_*C*H*_2_*NH), 37.54 (*C*-β), 31.9, 29.67, 29.63, 29.59, 29.53, 29.41, 29.33, 29.22 (each *C*H_2_), 28.30 (COC(CH_3_)_3_), 26.83, 22.67 (each *C*H_2_), 20.82–20.57 (overlap of O(CO)*C*H_3_), 14.93 (NHC_13_H_26_*C*H_3_); HRMS (MS-TOF): [M+H]^+^ calcd. for C_39_ H_67_N_3_O_14_: 801.4623 found 801.4613.

*N^4^-[2-O-(2,3,4,6-Tetra-O-acetyl-β-D-galactopyranosyl)-ethyl]-N^2^-decanoyl-L-asparagine tetradecylamide *(**11b**). A solution of **13** (0.11 g, 0.13mmol) in anhydrous CH_2_Cl_2_, (6 mL) was cooled in an ice bath and TFA (0.15 mL, 1.37 mmol) was added. The reaction mixture was heated to 50 °C for 1.5 h. The organic solvent was removed *in vacuo* and the residue obtained was diluted with ethyl acetate and washed with aqueous sat. NaHCO_3_ solution and brine, dried (MgSO_4_) and concentrated to yield the corresponding deprotected amine as a brown oil, which was used without further purification (0.071 g, 74%). ^1^H-NMR (300 MHz, CDCl_3_): δ 7.41 (bs, 1 H, CON*H*C_14_CH_29_), 6.42 (t, *J =* 5.1 Hz, 1 H, CH_2_CH_2_N*H*CO), 5.38 (d, *J* = 2.4 Hz, 1 H, *H*-4), 5.16 (dd, *J = 7*.8 Hz, *J =* 10.5 Hz, 1 H, *H*-2), 5.01 (dd, *J =* 3.3 Hz, *J =* 10.5 Hz, 1 H, *H*-3), 4.48 (d, *J =* 7.8 Hz, 1 H, *H*-1), 4.2-4.1 (m, 2 H, overlap of *H*-6, *H*-6'), 3.94-3.89 (m, 1 H, *H*-5) 3.87-3.82 (m,1 H, 1 H of OC*H*_2_CH*_2_*NH), 3.67-3.64 (m, 2 H, overlap of 1 H of OC*H*_2_CH*_2_*NH, H-α), 3.42-3.46 (bm, 2 H, OCH_2_C*H*_2_NH), 3.20-3.22 (m, 2 H, NHC*H*_2_C_13_H_27_),*2*.68 (dd, *J =* 3.9 Hz, *J =* 15 Hz, 1 H, H-β), 2.46 (dd, *J =* 7.8 Hz, *J =* 15 Hz, 1 H, H-β'), 2.15, 2.07, 2.03, 1.97 (each s, 3 H, O(CO)C*H*_3_), 1.50-1.43 (m, 2 H, NHCH_2_C*H*_2_C_12_H_25_), 1.23 (bs, 22 H, NHC_2_H_4_(C*H*_2_)_11_CH_3_), 0.87-0.83 (t, *J =* 6.6 Hz, 3 H, NHC_13_H_26_C*H*_3_); HRMS (MS-TOF): [M+H]^+^ calcd. for C_34_H_59_N_3_O_12_: 701.4099 found 701.4088. HOBt (0.041 g, 0.3 mmol) was added to a stirring solution of decanoic acid (0.048 g, 0.27 mmol) and TBTU (0.098 g, 0.3 mmol) dissolved in anhydrous DMF (6 mL), under N_2_ at rt. It was stirred for 10 min and the amine obtained from **13** as described above (0.06 g, 0.28 mmol) was dissolved in anhydrous DMF (8 mL) and added slowly. It was stirred for 18 h. The reaction mixture was concentrated *in vacuo*, diluted with ethyl acetate, washed with brine, dried (MgSO_4_) and concentrated . The residue obtained was purified by flash chromotagraphy (ethyl acetate) to afford **11b** as a white solid (0.15 g, 63%). [α]^22^_D_ = +5.8 (c 0.8, CH_2_Cl_2_,); IR (NaCl film): 3289.5, 3098.3, 2919.3, 2850.8, 1750.8, 168.1, 1646.5, 1542.4, 1467.4, 1370.4, 1225.5, 1174.9, 1058.5 cm^−1^; ^1^H-NMR (300 MHz, CDCl_3_): δ 7.43 (d, *J =* 6.9 Hz, 1 H, N*H*COC_9_H_19_), 7.08 (t, *J =* 5.4 Hz, 1 H, CON*H*C_14_CH_29_), 6.26 (t, *J =* 5.4 Hz, 1 H, CH_2_CH_2_N*H*CO), 5.40 (d, *J =* 2.4 Hz, 1 H, *H*-4), 5.18 (dd, *J = 7*.8 Hz, *J =* 10.5 Hz, 1 H, *H*-2), 5.04 (dd, *J =* 3.3 Hz, *J =* 10.5 Hz, 1 H, *H*-3), 4.70–4.64 (m, 1 H, *H*-α), 4.54 (d, *J =* 7.8 Hz, 1 H, *H*-1), 4.22–4.11 (m, 2 H, overlap of *H*-6, *H*-6'), 3.97–3.94 (m, 1 H, *H*-5) 3.92–3.85 (m, 1 H, 1 H of OC*H*_2_CH*_2_*NH), 3.69–3.64 (m, 1 H, 1 H of OC*H*_2_CH*_2_*NH), 3.57–3.39 (m, 2 H, OCH_2_C*H*_2_NH), 3.22–3.16 (m, 2 H, NHC*H*_2_C_13_H_27_),*2*.81 (dd, *J =* 3.3 Hz, *J =* 15.3 Hz, 1 H, H-β), 2.46 (dd, *J =* 6.9, *J =* 15.6, 1 H, H-β'), 2.25–2.20 (t, *J =* 7.5 Hz, 2 H, COC*H*_2_C_8_H_17_), *2*.16, 2.09, 2.05, 1.9 (each s, 3 H, O(CO)C*H*_3_), 1.62–1.60 (m, 2 H, COCH_2_C*H*_2_C_7_H_15_), 1.46–1.45 (m, 2 H, NHCH_2_C*H*_2_C_12_H_25_), 1.26 (bs, 34 H, overlap of COC_2_H_4_(C*H*_2_)_6_CH_3_, NHC_2_H_4_(C*H*_2_)_11_CH_3_), 0.90–0.85 (t, *J =* 6.6 Hz, 6 H, overlap of COC_8_H_16_C*H*_3_, NHC_13_H_26_C*H*_3_); ^13^C-NMR (75 Hz, CDCl_3_): δ 173.67, 171.68, 170.61, 170.38, 170.20, 170.06, 169.90 (each *C*O), 101.33 (*C*-1), 70.81, 70.73 (*C*-5, *C*-3), 68.97. (*C*-2), 68.51 (NH*C*H_2_C_13_H_27_), 66.98 (*C*-4), 61.30 (*C*-6), 49.72 (*C*-α), 39.67, 39.31 (each O*C*H_2_*C*H*_2_*NH), 36.98 (*C*-β), 36.62, 31.92, 29.70, 29.65, 29.56, 29.52, 29.36, 29.29, 28.31, 26.88, 25.63, 22.62 (each *C*H_2_), 20.87–20.85 (overlap of O(CO)*C*H_3_), 14.12 (overlap of COC_8_H_16_*C*H_3_, NHC_13_H_26_*C*H_3_); HRMS (MS-TOF): [M+H]^+^ calcd. for C_44_H_77_N_3_O_13_: 855.5456 found 855.5492.

*N^4^-[2-O-(2,3,4,6-Tetra-O-acetyl-β-D-galactopyranosyl)-ethyl]-N^2^-tetracosanoyl-L-asparagine tetradecylamide *(**14**). HOBt (0.022 g, 0.16 mmol) was added to a stirring solution of tetracosanoic acid (0.056 g, 0.15 mmol) and TBTU (0.056 g, 0.16 mmol) dissolved in anhydrous DMF (6 mL), under N_2_ at rt. It was stirred for 10 min and the amine obtained from **13** as described above (0.128 g, 0.18 mmol) was dissolved in anhydrous DMF (8 mL) and added slowly. It was stirred for 3 h. The reaction mixture was concentrated *in vacuo*, diluted with ethyl acetate, washed with brine, dried (MgSO_4_) and concentrated. The residue obtained was purified by flash chromotagraphy (ethyl acetate) to afford **14** as a white solid, (0.030 g, 18%). [α]^22^_D_ = +3.8 (c 0.83, CH_2_Cl_2_,); IR (NaCl film): 3423.0, 2918.4, 2850.3, 1749.6, 1644.4,1543.1, 1465.6, 1369.9, 1223.4, 1058.3 cm^−1^; ^1^H-NMR (300 MHz, CDCl_3_): δ 7.44 (d, *J =* 6.9 Hz, 1 H, N*H*COC_23_H_47_), 7.09 (t, *J =* 4.8 Hz, 1 H, CON*H*C_14_CH_29_) 6.26 (bs, 1 H, CH_2_CH_2_N*H*CO), 5.40 (d, *J =* 3 Hz, 1 H, *H*-4), 5.17 (dd, *J = 7*.8 Hz , *J =* 10.5 Hz, 1 H, *H*-2), 5.04 (dd, *J =* 2.7 Hz, *J =* 10.2 Hz, 1 H, *H*-3), 4.70-4.64 (m, 1 H, *H*-α), 4.53 (d, *J =* 7.8 Hz, 1 H, *H*-1), 4.21-4.10 (m, 2 H, overlap of *H*-6, *H*-6'), 3.97–3.92 (tm, 1 H, H-5) 3.90–3.85 (m,1 H, 1 H of OC*H*_2_CH*_2_*NH), 3.71–3.64 (m, 1 H, 1 H of OC*H*_2_CH*_2_*NH), 3.54–3.44 (m, 2 H, OCH_2_C*H*_2_NH), 3.22–3.15 (m, 2 H, NHC*H*_2_C_13_H_27_),2.77 (dd, *J =* 3.3 Hz, *J =* 15.3 Hz, 1 H, H-β), 2.45 (dd, *J =* 6.9 Hz, *J =* 15.6 Hz, 1 H, H-β'), 2.25–2.20 (t, *J =* 7.5 Hz, 2 H, COC*H*_2_C_22_ H_45_), *2*.16, 2.09, 2.04, 1.90 (each s, 3 H, O(CO)C*H*_3_), 1.62–1.59 (m, 2 H, COCH_2_C*H*_2_C_21_H_43_), 1.46–1.45 (m, 2 H, NHCH_2_C*H*_2_C_12_H_25_), 1.25 (bs, 62 H, overlap of COC_2_H_4_(C*H*_2_)_20_CH_3_, NHC_2_H_4_(C*H*_2_)_11_CH_3_), 0.89–0.85 (t, *J =* 6.3 Hz, 6 H, overlap of COC_22_H_44_C*H*_3_, NHC_13_H_26_C*H*_3_); ^13^C-NMR (75 Hz, CDCl_3_): δ 173.57, 171.72, 170.58, 170.38, 170.20, 170.07, 169.90 (each *C*O), 101.34 (*C*-1), 70.82, 70.73 (*C*-5, *C*-3), 68.98. (*C*-2), 68.54 (NH*C*H_2_C_13_H_27_), 66.98 (*C*-4), 61.31 (*C*-6), 49.72 (*C*-α), 39.66, 39.24 (each O*C*H_2_*C*H*_2_*NH), 36.95 (*C*-β), 36.64 (CO*C*H_2_(CH_2_)_20_CH_3_), 31.92, 31.86, 29.66,29.56, 29.45, 29.36, 29.28, 26.89, 22.69, 22.66 (each *C*H_2_), 20.88 (overlap of O(CO)*C*H_3_), 14.12 (overlap of COC_22_H_44_*C*H_3_, NHC_13_H_26_*C*H_3_); HRMS (MS-TOF): [M+H]^+^ calcd. for C_58_H_105_N_3_O_13_: 1052.772 found 1052.775.

*N^4^-[2-O-(β-D-Galactopyranosyl)-ethyl]-N^2^-decanoyl-L-asparagine tetradecylamide *(**1**). Triethylamine (0.1 mL) was added to a stirring solution of **11b** (0.120 g, 0.14 mmol) dissolved in CH_2_Cl_2_/MeOH/H_2_O (3 mL/6 mL/3 mL) at 40 °C. It was stirred for 18 h. The reaction mixture was concentrated under reduced pressure to afford **1** as a white solid (0.080 g, 83%). [α]^22^_D_= −6.0 (c 0.33, C_5_H_5_N); ^1^H-NMR (300 MHz, *d*_5_-Pyr): δ 8.95 (d, *J =* 8.1 Hz, 1 H, N*H*COC_9_H_19_), 8.87 (t, *J =* 5.4 Hz, 1 H, CH_2_CH_2_N*H*CO), 8.55 (t, *J =* 5.7 Hz, 1 H, CON*H*C_14_CH_29_), 7.05, 6.79, 6.63, 6.39 (each bs, 1 H, O*H*), 5.55–5.53 (m, 1 H, *H*-α), 4.79 (d, *J =* 7.5 Hz, 1 H, *H*-1), 4.51–4.33 (m, 4 H, overlap of *H*-2, *H*-4, *H*-6, *H*-6'), 4.18–4.07 (m, 3 H, overlap of *H*-3, *H*-5, 1 H of OC*H*_2_CH*_2_*NH), 3.99–3.95 (m, 1 H, 1 H of OC*H*_2_CH*_2_*NH), 3.77–3.65 (m, 2 H, OCH_2_C*H*_2_NH), 3.22–3.16 (m, 2 H, NHC*H*_2_C_13_H_27_),3.18 (td, *J =* 6.6 Hz, *J =* 1.2 Hz, 2 H, overlap of *H*-β, *H*-β'), 2.39–2.34 (t, *J =* 7.5 Hz, 2 H, COC*H*_2_C_8_H_17_), 1.79–1.69 (m, 2 H, COCH_2_C*H*_2_C_7_H_15_), 1.60–1.50 (m, 2 H, NHCH_2_C*H*_2_C_12_H_25_), 1.20 (bs, 34 H, overlap of COC_2_H_4_(C*H*_2_)_6_CH_3_, NHC_2_H_4_(C*H*_2_)_11_CH_3_), 0.89–0.82 (m, 6 H, overlap of COC_8_H_16_C*H*_3_, NHC_13_H_26_C*H*_3_); ^13^C-NMR (75 Hz, *d*_5_-Pyr): δ 175.40, 173.98, 173.04 (each *C*O), 107.68 (*C*-1), 78.96, 77.22, 74.52, 72.25 (*C*-2, *C*-4, *C*-3, *C*-5), 71.68 (NH*C*H_2_C_13_H_27_), 64.53 (*C*-6), 53.27 (*C*-α), 42.50, 41.80 (each O*C*H_2_*C*H*_2_*NH), 40.71 (*C*-β), 38.49, 38.49, 34.09, 34.01,32.04, 31.95, 31.90, 31.68, 31.66, 31.63, 31.58, 31.49, 29.22, 28.09 (each *C*H_2_), 18.63 (overlap of COC_8_H_16_*C*H_3_, NHC_13_H_26_*C*H_3_); HRMS (MS-TOF): [M+H]^+^ calcd. for C_36_H_69_N_3_O_9_: 688.5107 found 688.5099.

*N^4^-[2-O-(β-D-Galactopyranosyl)-ethyl]-N^2^-tetracosanoyl-L-asparagine tetradecylamide *(**2**). Triethylamine (0.1 mL) was added to a stirring solution of **14** (0.016 g, 0.015mmol) dissolved in CH_2_Cl_2_/MeOH/H_2_O/THF (1 mL/2 mL/1 mL/2 mL) at 40 °C. The reaction mixture was stirred and its progress was followed by ^1^H-NMR spectra of aliquots. The reaction was deemed complete after 36 h. The reaction was concentrated under reduced pressure to afford **2** as a white solid (0.05 g, 38%). ^1^H-NMR (300 MHz, *d*_5_-Pyr): δ 9.02–8.96 (m, 1 H, N*H*COC_23_H_47_), 8.89–8.79 (m, 1 H, CH_2_CH_2_N*H*CO), 8.54–8.52 (m, 1 H, CON*H*C_14_CH_29_), 5.54 (dd, *J* = 6.3, 12.9 Hz, 1 H, *H*-α), 4.80 (d, *J* = 7.8 Hz, 1 H, *H*-1), 4.52–4.36 (m, 4 H, overlap of *H*-2, *H*-4, *H*-6,*H*-6'), 4.18–4.10 (m, 3 H, overlap of *H*-3, *H*-5, 1 H of OC*H*_2_CH*_2_*NH), 3.99–3.95 (m, 1 H, 1 H of OC*H*_2_CH*_2_*NH), 3.77–3.65 (m, 2 H, OCH_2_C*H*_2_NH), 3.48–3.38 (m, 2 H, NHC*H*_2_C_13_H_27_), 3.18 (m, 2 H, *H*-β, *H*-β'), 2.40–2.35 (m, 2 H, COC*H*_2_C_22_H_45_), 1.79–1.69 (m, 2 H, COCH_2_C*H*_2_C_21_H_43_), 1.60–1.50 (m, 2 H, NHCH_2_C*H*_2_C_12_H_25_), 1.20 (bs, 62 H, overlap of COC_2_H_4_(C*H*_2_)_20_CH_3_, NHC_2_H_4_(C*H*_2_)_11_CH_3_), 0.89–0.82 (m, 6 H, overlap of COC_22_H_44_C*H*_3_, NHC_13_H_26_C*H*_3_); HRMS (MS-TOF): [M+H]^+^ calcd. for C_50_H_97_N_3_O_9_: 883.7225 found 883.7278.

*N^4^-(2,3,4,6-Tetra-O-acetyl-β-D-galactopyranosyl)-N^2^-tert-butoxycarbonyl-L-asparagine benzyl ester *(**15**) [30]. HOBt (1.30 g, 9.60 mmol) was added to a stirring solution of *N*-Boc-L-Asp-OBn **8** (1.55 g, 4.80 mmol) and TBTU (3.08 g, 0.720 mmol) in anhydrous DMF (25 mL) under N_2 _at rt. It was stirred for 30 min and galactosyl amine **5 **(2 g, 5.76 mmol) dissolved in anhydrous DMF (10 mL) was added dropwise to the solution. It was stirred for 18 h. The reaction mixture was concentrated *in vacuo*, diluted with ethyl acetate, washed with water, HCl 0.1 N and aqueous sat. NaHCO_3_solution, dried (MgSO_4_) and concentrated. Flash chromatography (hexane/ethyl acetate 1:1) afforded **15** as a white solid (1.70 g, 72%). This was used without further purification. A small sample of **15** was recrystallised in CHCl_3_/hexane to give white crystals used for characterisation. [α]^22^_D_ = +30 (c 1.2, CHCl_3_); m.p. = 148–150 °C; IR (NaCl film): 3348.7, 2965.2, 1749.6, 1499.7, 1369.0, 1221.7, 1054.5 771.3 cm^−1^; ^1^H-NMR (300 MHz, CDCl_3_): δ 7.34–7.33 (m, 5 H, Ph-*H*), 6.38 (d, *J* = 9 Hz, N*H*COC(CH_3_)_3_), 5.70 (d, *J* = 9 Hz,1 H, N*H*COCH_2_), 5.43 (d, *J* = 3 Hz, 1 H, *H*-4), 5.21–5.04 (m, 5 H, overlap of CH_2_Ph, *H*-1, *H*-2, *H*-3), 4.58 (t, *J* = 6 Hz, 1 H, *H*-α), 4.15–3.97 (m, 3 H, overlap of *H*-5, *H*-6, *H*-6'), 2.95–2.84 (m, 1 H, *H*-β'), 2.71 (dd, *J* = 3 Hz, *J *= 15 Hz, 1 *H*, H-β), 2.13 , 2.03,1.99, 1.98 (each s, 3 H, O(CO)C*H*_3_), 1.41 (9 H, O(CO)C*H*_3_)_3_); ^13^C-NMR (75 MHz, CDCl_3_): δ171.53, 171.12, 170.54, 170.33, 169.95, 169.74 (each *C*O), 135.33 (*C*-Ph), 128.50, 128.26, 127.88 (*C*H-Ph), 80.10 (*C*(CH_3_)_3_), 78.44 (*C*-1), 72.40 (*C*-5), 70.67 (*C*-3), 68.17 (*C*-2), 67.29 (CH_2_Ph), 67.06 (*C*-4), 61.08 (*C*-6), 50.09 (*C*-α), 37.85 (*C*-β), 28.25 (C(*C*H_3_)_3_), 20.66, 20.59, 20.57, 20.52 (each O(CO)*C*H_3_); HRMS (MS-TOF): [M+H]^+^ calcd. for C_30_H_41_N_2_O_14_: 653.2552 found 653.2541.

*N^4^-(2,3,4,6-Tetra-O-acetyl-β-D-galactopyranosyl)-N^2^-decanoyl-L-asparagine benzyl ester *(**16**). TFA (1.34 mL, 17.93 mmol) in anhydrous CH_2_Cl_2_ (1.34 mL) was added dropwise to a solution of **15** (1.17 g, 1.79 mmol) in anhydrous CH_2_Cl_2_ (10 mL). It was stirred for 6 h. The reaction mixture was concentrated in the rotary evaporator and the residue was dissolved in CH_2_Cl_2_ and washed with aqueous sat. NaHCO_3_ solution, brine and water. The organic phase was dried (MgSO_4_) and concentrated *in vacuo* to yield the corresponding amine as a white foam (0.73 g, 74%). The compound was used without further purification. ^1^H-NMR (300 MHz, CDCl_3_): δ 8.10 (d, *J *= 9.3 Hz, 1 H, N*H*COCH_2_), 7.51–7.31 (m, 5 H, Ph-*H*), 5.40 (d, *J *= 1.4 Hz, 1 H, *H*-4), 5.22 (t, *J *= 9.3 Hz, 1 H, *H*-1), 5.12–5.07 (m, 4 H, overlap of CH_2_Ph, *H*-2, *H*-3), 4.11–3.97 (m, 3 H, overlap of *H*-5, *H*-6, *H*-6'), 3.67 (bs, 1 H, *H*-α), 2.67–2.63 (m, 1 H, *H*-β), 2.39 (dd, *J *= 9.6 Hz, *J* = 5.3 Hz, 1 H, *H*-β'), 2.10, 2.00, 1.99, 1.95 (each s, 3 H, O(CO)C*H*_3_); HRMS (MS-TOF): [M+Na]^+^ calcd. for C_25_H_32_O_12_N_2_Na: 553.2028 found 553.2024. TBTU (56 mg, 0.18 mmol) and HOBt (24 mg, 0.18 mmol) were added to a solution of decanoic acid (27 mg, 0.16 mmol) in anhydrous DMF (2 mL) under N_2_ at rt. It was stirred for 20 min and the free amine obtained from **15** as described above (88 mg, 0.16 mmol) in anhydrous DMF (1 mL) was added dropwise to the solution. It was stirred for 18 h. The reaction mixture was concentrated *in vacuo*, diluted with ethyl acetate, washed with water and brine, dried (MgSO_4_) and concentrated. The residue obtained was purified by flash chromotagraphy (hexane/ethyl acetate 1:1) to yield **16** as a colourless oil (94 mg, 85%). [α]^22^_D_ = +27.2 (c 1.76, CHCl_3_); IR (NaCl film): 3330.9, 2926.4, 1751.0, 1674.3, 1530.8, 1370.1, 1222.5, 1179.4, 1052.8, 698.5 cm^−1^; ^1^H-NMR (300 MHz, CDCl_3_): δ 7.33–7.31 (m, 5 H, Ph-*H*), 6.73 (d, *J* = 8.3 Hz, 1 H, N*H*COC_9_H_19_), 6.50 (d, *J *= 8.7 Hz, 1 H, N*H*COCH_2_), 5.43 (d, *J *= 2.0 Hz, 1 H, *H*-4), 5.20–5.04 (m, 5 H, overlap of *H*-1, *H*-2, *H*-3, C*H*_2_Ph), 4.93–4.87 (m, 1 H, *H*-α), 4.15–3.97 (m, 3 H, overlap of *H*-5, *H*-6, *H*-6'), 2.90 (dd, *J* = 4.1 Hz, *J* = 16.5 Hz, 1 H, *H*-β), 2.70 (dd, *J* = 4.4 Hz, *J* = 16.4 Hz, 1 H, *H*-β'), 2.22–2.17 (t, *J *= 7.3 Hz, 2 H, COC*H*_2_C_8_H_17_), 2.13, 2.03, 1.99, 1.98 (each s, 3 H, O(CO)C*H*_3_), 1.61–1.56 (m, 2 H, COCH_2_C*H*_2_C_7_H_15_), 1.24 (bs, 12 H, COC_2_H_4_(C*H*_2_)_6_CH_3_), 0.88–0.84 (t, *J* = 7.0 Hz, 3 H, COC_8_H_16_C*H*_3_); ^13^C-NMR (75 MHz, CDCl_3_): δ 173.06, 171.49, 170.90, 170.82, 170.28, 169.95, 169.76 (each *C*O), 135.23, (Ph-*C*), 128.53, 128.33, 127.92 (Ph-*C*H), 78.44 (*C*-1), 72.37 (*C*-5), 70.66 (*C*-3), 68.11 (*C*-2), 67.36 (*C*H_2_Ph), 67.00 (*C*-4), 61.00 (*C*-6), 48.42 (*C*-α), 37.43 (*C*-β), 36.49, 31.81, 29.36, 29.27, 29.22, 29.18, 25.49, 22.62 (each *C*H_2_), 20.62, 20.55, 20.53, 20.49 (each O(CO)*C*H_3_), 14.06 (COC_8_H_16_*C*H_3_); HRMS (MS-TOF,): [M+Na]^+^ calcd. for C_35_H_50_O_13_N_2_Na: 707.3386 found 707.3376.

*N^4^-(2,3,4,6-Tetra-O-acetyl-β-D-galactopyranosyl)-N^2^-tetracosanoyl-L-asparagine benzyl ester *(**17**). TBTU (59 mg, 0.183 mmol) and HOBt (25 mg, 0.183 mmol) were added to tetracosanoic acid (62 mg, 0.167 mmol) in anhydrous DMF (3 mL) containing 4 Å MS under N_2_ at rt. It was stirred for 30 min and the free amine obtained from **15** as described above (88 mg, 0.16 mmol) in anhydrous DMF (2 mL) was added dropwise to the solution. It was stirred for 2 h at 50 °C. The reaction mixture was concentrated *in vacuo*, diluted with ethyl acetate, washed with water and brine, dried (MgSO_4_) and concentrated under reduced pressure. The residue obtained was purified by flash chromotagraphy (hexane/ethyl acetate 1:1) to yield **17** as a white solid (93 mg, 62%). [α]^25^_D_ = +18.9 (c 0.95, ethyl acetate); IR (NaCl film): 2918.7, 2850.5, 1750.5, 1371.1, 1231.8, 1054.9, 913.2, 743.7 cm^−1^; ^1^H-NMR (300 MHz, CDCl_3_): δ 7.33–7.31 (m, 5 H, *H*-Ph), 6.73 (d, *J* = 8.3 Hz, 1 H, N*H*COC_23_H_47_), 6.48 (d, *J *= 8.6 Hz, 1 H, N*H*COCH_2_), 5.43 (d, *J *= 2.0 Hz, 1 H, *H*-4), 5.20–5.07 (m, 5 H, overlap of *H*-1, *H*-2, *H*-3, C*H*_2_Ph), 4.93–4.88 (m, 1 H, *H*-α), 4.15–3.97 (m, 3 H, overlap of *H*-5, *H*-6, *H*-6'), 2.91 (dd, *J* = 3.9 Hz, *J* = 16.4 Hz, 1 H, *H*-β'), 2.71 (dd, *J* = 4.4 Hz, *J* = 16.4 Hz, 1 H, *H*-β'), 2.22–2.17 (t, *J *= 7.5 Hz, 2 H, COC*H*_2_C_22_H_45_), 2.13, 2.04, 2.03, 1.99 (each s, 3 H, O(CO)C*H*_3_), 1.61–1.56 (m, 2 H, COCH_2_C*H*_2_C_21_H_43_), 1.25 (bs, 40 H, COC_2_H_4_(C*H*_2_)_20_CH_3_, 0.89–0.85 (t, *J* = 7.0 Hz, 3 H, COC_22_H_44_C*H*_3_); ^13^C-NMR (75 Hz, CDCl_3_): δ 173.09, 171.53, 170.91, 170.82, 170.29, 169.97, 169.77 (each *C*O), 135.23, 128.54, 128.35 (*C*H-Ph), 127.93 (*C*-Ph), 78.46 (*C*-1), 72.38 (*C*-5), 70.66 (*C*-3), 68.12 (*C*-2), 67.39 (CH_2_Ph), 67.00 (*C*-4), 61.00 (*C*-6), 48.43 (*C*-α), 37.45 (*C*-β), 36.52, 31.89, 29.67, 29.62, 29.45, 29.33, 29.31, 29.21, 25.51, 22.66 (each *C*H_2_), 20.64, 20.57, 20.55, 20.51 (each O(CO)*C*H_3_), 14.09 (COC_22_H_44_*C*H_3_); HRMS (MS-TOF): [M+H]^+^ calcd. for C_49 _H_79_O_13_N_2_: 904.5610 found 904.5632.

*N^4^-(2,3,4,6-Tetra-O-acetyl-β-D-galactopyranosyl)-N^2^-decanoyl-L-asparagine tetradecylamide *(**18**). H_2_ gas was bubbled through a suspension of **16** (56 mg, 0.079 mmol) in ethyl acetate (10 mL) and Pd/C 10% w/w (6 mg, 10% w/w) was added. It was left to stir for 3 h and then the reaction mixture was filtered through Celite washing with ethyl acetate and concentrated *in vacuo*, to yield the corresponding carboxylic acid as a colourless oil, which was used without further purification (30 mg, 61%). ^1^H-NMR (300 MHz, CDCl_3_): δ 7.24 (d, *J* = 6.8 Hz, 1 H, N*H*COC_9_H_19_), 6.57 (d, *J *= 9.4 Hz, 1 H, N*H*COCH_2_), 5.53 (d, *J *= 1.7 Hz, 1 H, *H*-4), 5.35 (m, 1 H, *H*-1), 5.16–5.10 (m, 2 H, overlap of *H*-2, *H*-3), 4.74–4.71 (m, 1 H, *H*-α), 4.25–4.00 (m, 3 H, overlap of *H*-5, *H*-6, *H*-6'), 2.87 (dd, *J* = 3.6 Hz, *J* = 16.4 Hz, 1 H, *H*-β), 2.77 (dd, *J* = 4.8 Hz, *J* = 16.5 Hz, 1 H, *H*-β'), 2.32–2.27 (t, *J *= 8.0 Hz, 2 H, COC*H*_2_C_8_H_17_), 2.15 (s, 3 H, O(CO)*C*H_3_), 2.05 (s, 6 H, O(CO)C*H*_3_ × 2), 2.00 (s, 3 H, O(CO)C*H*_3_), 1.65–1.60 (m, 2 H, COCH_2_C*H*_2_C_7_H_15_), 1.25 (bs, 12 H, COC_2_H_4_(C*H*_2_)_6_CH_3_), 0.89–0.85 (t, *J* = 6.9 Hz, 3 H, COC_8_H_16_C*H*_3_); HRMS (MS-TOF): [M+H]^+^ calcd. for C_28_H_45_O_13_N_2_: 617.2916 found 617.2900. TBTU (14 mg, 0.045 mmol) and HOBt (6 mg, 0.045 mmol) were added to a solution of the carboxylic acid obtained from **16** as described above (25 mg, 0.041 mmol) in anhydrous DMF (3 mL) under N_2_ at rt. It was stirred for 30 min and tetradecylamine (9 mg, 0.041 mmol) was added to the solution and it was stirred for 3 h. The reaction mixture was concentrated *in vacuo*, diluted with ethyl acetate, washed with brine and water, dried (MgSO_4_) and concentrated. The residue obtained was purified by flash chromotagraphy (hexane/ethyl acetate 1:1) to yield **18** as a white solid (18 mg, 55%). [α]^25^_D_ = +20.0 (c 0.75, CH_2_Cl_2_); IR (NaCl film): 3286.1, 2924.1, 2853.8, 1751.2, 1642.6, 1546.1, 1466.7, 1371.0, 1227.6, 1054.9 cm^−1^; ^1^H-NMR (300 MHz, CDCl_3_): δ 7.58 (d, *J* = 7.6 Hz, 1 H, N*H*COC_9_H_19_), 6.79–6.74 (m, 2 H, overlap of N*H*COCH_2_, CON*H*C_14_CH_29_), 5.44 (d, *J *= 1.6 Hz, 1 H, *H*-4), 5.23–5.10 (m, 3 H, overlap of *H*-1, *H*-2, *H*-3), 4.71–4.67 (m, 1 H, *H*-α), 4.16–3.99 (m, 3 H, overlap of *H*-5, *H*-6, *H*-6'), 3.18–3.11 (m, 2 H, NHC*H*_2_C_13_H_27_), 2.69 (dd, *J* = 3.4 Hz, *J* = 15.6 Hz, 1 H, *H*-β), 2.44 (dd, *J* = 5.6 Hz, *J* = 15.5 Hz, 1 H, *H*-β'), 2.25–2.21 (m, 2 H, COC*H*_2_C_8_H_17_), 2.17, 2.14, 2.04, 2.00 (each s, 3 H, O(CO)C*H*_3_), 1.65 (bs, 2 H, COCH_2_C*H*_2_C_7_H_15_), 1.46–1.42 (m, 2 H, NHCH_2_C*H*_2_C_12_H_25_), 1.25 (bs, 34 H, overlap of COC_2_H_4_(C*H*_2_)_6_CH_3_, NHC_2_H_4_(C*H*_2_)_11_CH_3_), 0.90–0.85 (t, *J* = 6.9 Hz, 6 H, overlap of COC_8_H_16_C*H*_3_, NHC_13_H_26_C*H*_3_); ^13^C-NMR (75 MHz, CDCl_3_): δ 173.77, 173.03, 172.25, 170.35, 170.30, 170.01, 169.79 (each *C*O), 78.49 (*C*-1), 72.31 (*C*-5), 70.73 (*C*-3), 67.84 (*C*-2), 67.05 (*C*-4), 61.06 (*C*-6), 49.75 (*C*-α), 39.59 (NH*C*H_2_C_13_H_27_), 36.60 (*C*-β), 36.21, 31.91, 31.84, 29.68, 29.65, 29.61, 29.55, 29.44, 29.35, 29.31, 29.26, 26.89, 25.63, 22.68, 22.65 (each *C*H_2_), 20.89, 20.67, 20.59, 20.54, (each O(CO)*C*H_3_), 14.11 (overlap of COC_8_H_16_*C*H_3_, NHC_13_H_26_*C*H_3_); HRMS (MS-TOF): [M+Na]^+^ calcd. for C_42_ H_73_O_12_N_3_Na: 834.5086 found 834.5079.

*N^4^-(2,3,4,6-Tetra-O-acetyl-β-D-galactopyranosyl)-N^2^-tetracosanoyl-L-asparagine tetradecylamide *(**19**). H_2_ gas was bubbled through a suspension of **17** (67 mg, 0.074 mmol) in ethyl acetate (5 mL) and Pd/C 10% w/w (7 mg, 10% w/w) was added. It was left to stir for 18 h and then the reaction mixture was filtered through Celite, washed with ethyl acetate and concentrated *in vacuo* to yield the corresponding carboxylic acid as a white solid, which was used without further purification (60 mg, 55%). ^1^H-NMR (300 MHz, CDCl_3_): δ 7.20 (d, *J* = 7.0 Hz, 1 H, N*H*COC_23_H_47_), 6.62 (d, *J *= 9.2 Hz, 1 H, N*H*COCH_2_), 5.51 (d, *J *= 1.2 Hz, 1 H, *H*-4), 5.34–5.28 (m, 1 H, *H*-1), 5.12–5.10 (m, 2 H, overlap of *H*-2, *H*-3), 4.76–4.71 (m, 1 H, *H*-α), 4.22–4.02 (m, 3 H, overlap of *H*-5, *H*-6, *H*-6'), 2.96–2.84 (m, 1 H, *H*-β), 2.75 (dd, *J* = 5.0 Hz, *J* = 16.5 Hz, 1 H, *H*-β'), 2.37–2.26 (m, 2 H, COC*H*_2_C_22_H_45_), 2.15, 2.06, 2.05, 2.00 (each s, 3 H, O(CO)C*H*_3_), 1.67–1.58 (m, 2 H, COCH_2_C*H*_2_C_21_H_43_), 1.25 (bs, 40 H, COC_2_H_4_(C*H*_2_)_20_CH_3_), 0.90–0.85 (t, *J* = 6.9 Hz, 3 H, COC_22_H_44_C*H*_3_); HRMS (MS-TOF): [M+H]^+^ calcd. for C_42_H_73_O_13_N_2_: 813.5107 found 813.5106. TBTU (12 mg, 0.037 mmol) and HOBt (5 mg, 0.037 mmol) were added to a solution of the carboxylic acid obtained from **17** as described above (27 mg, 0.033 mmol) in anhydrous DMF (3 mL), containing 4 Å MS, under N_2 _and at rt. It was stirred for 20 min and tetradecylamine (7 mg, 0.033 mmol) was added to the solution. It was stirred for 18 h. The reaction mixture was concentrated *in vacuo*, diluted with ethyl acetate, washed with brine and water, dried (MgSO_4_) and concentrated under reduced pressure. Flash chromatography (hexane/ethyl acetate 1:1) afforded **19** as a white solid (15 mg, 45%). [α]^25^_D_ = +09.2 (c 0.65, CH_2_Cl_2_); IR (NaCl film): 3426.0, 2918.5, 2850.5, 1750.7, 1641.8, 1228.5 cm^−1^; ^1^H-NMR (300 MHz, CDCl_3_): δ 7.58 (d, *J* = 7.8 Hz, 1 H, N*H*COC_23_H_47_), 6.78–6.73 (m, 2 H, overlap of N*H*COCH_2_, CON*H*C_14_CH_29_), 5.45 (d, *J *= 1.8 Hz, 1 H, *H*-4), 5.26–5.10 (m, 3 H, overlap of *H*-1, *H*-2, *H*-3), 4.71–4.66 (m, 1 H, *H*-α), 4.17–3.98 (m, 3 H, overlap of *H*-5, *H*-6, *H*-6'), 3.26–3.11 (m, 2 H, NHC*H*_2_C_13_H_27_), 2.69 (dd, *J* = 3.5 Hz, *J* = 15.7 Hz, 1 H, *H*-β), 2.44 (dd, *J* = 5.6 Hz, *J* = 15.7 Hz, 1 H, *H*-β'), 2.24–2.21 (m, 2 H, COC*H*_2_C_22_H_45_), 2.17, 2.14, 2.04, 2.00 (each s, 3 H, O(CO)C*H*_3_), 1.66 (bs, 2 H, COCH_2_C*H*_2_C_21_H_43_), 1.46–1.42 (m, 2 H, NHCH_2_C*H*_2_C_12_H_25_), 1.25 (bs, 62 H, overlap of COC_2_H_4_(C*H*_2_)_20_CH_3_, NHC_2_H_4_(C*H*_2_)_11_CH_3_), 0.90–0.85 (t, *J* = 6.9 Hz, 6 H, overlap of COC_22_H_44_C*H*_3_, NHC_13_H_26_C*H*_3_); ^13^C-NMR (75 MHz, CDCl_3_): δ 173.77, 173.03, 172.24, 170.35, 170.29, 170.00, 169.78 (each *C*O), 78.47 (*C*-1), 72.30 (*C*-5), 70.72 (*C*-3), 67.83 (*C*-2), 67.04 (*C*-4), 61.04 (*C*-6), 49.73 (*C*-α), 39.57 (NH*C*H_2_C_13_H_27_), 36.59 (*C*-β), 36.18,31.90, 29.68, 29.33, 26.87, 25.62, 22.66, (each *C*H_2_), 20.87, 20.64, 20.57, 20.51, (each O(CO)C*H*_3_), 14.09 (overlap of COC_22_H_44_*C*H_3_, NHC_13_H_26_*C*H_3_); HRMS (MS-TOF): [M+H]^+^ calcd. for C_56_H_102_O_12_N_3_: 1008.7458 found 1008.7429.

*N^4^-β-D-Galactopyranosyl-N^2^-decanoyl-L-asparagine tetradecylamide *(**3**). Triethylamine (0.05 mL) was added to a stirring solution of **18** (0.043 g, 0.053 mmol) dissolved in CH_2_Cl_2_/MeOH/H_2_O (1 mL/2 mL/1 mL) at 40 °C. It was stirred for 18 h. The precipitate formed was filtered through a vacuum to afford **3** as white crystals (7 mg, 21%). [α]^22^_D_ = −6.7 (c 0.6, C_5_H_5_N); ^1^H-NMR (300 MHz, *d*_5_-Pyr): δ 10.14 (d, *J *= 9.1 Hz, 1 H, N*H*COCH_2_), 8.94 (d, *J* = 7.9 Hz, 1 H, N*H*COC_9_H_19_), 8.53–8.48 (t, *J *= 5.7 Hz, 1 H, CON*H*C_14_CH_29_), 5.91–5.78 (t, *J* = 9.2 Hz, 1 H, *H*-1), 5.45 (dd, *J* = 6.8, 14.3 Hz, 1 H, *H*-α), 4.58 (d, *J* = 2.9 Hz, 1 H, *H*-4), 4.56–4.49 (t, *J* = 9.2 Hz, 1 H, *H*-2), 4.38 (dd, *J* = 2.1 Hz,*J* = 6.1 Hz, 2 H, *H*-6, *H*-6'), 4.17 (dd, *J *= 3.0 Hz, *J *= 9.0 Hz, 1 H, *H*-3), 4.13–4.09 (t, *J* = 5.8 Hz, 1 H, *H*-5), 3.45–3.34 (m, 2 H, NHC*H*_2_C_13_H_27_), 3.27 (d, *J* = 6.5 Hz, 2 H, *H*-β, *H*-β'), 2.36–2.31 (t, *J *= 7.5 Hz, 2 H, COC*H*_2_C_8_H_17_), 1.75–1.70 (m, 2 H, COCH_2_C*H*_2_C_7_H_15_), 1.58–1.50 (m, 2 H, NHCH_2_C*H*_2_C_12_H_25_), 1.24 (bs, 34 H, overlap of COC_2_H_4_(C*H*_2_)_6_CH_3_, NHC_2_H_4_(C*H*_2_)_11_CH_3_), 0.88–0.81 (m, 6 H, overlap of COC_8_H_16_C*H*_3, _NHC_13_H_26_C*H*_3_); ^13^C NMR (*d*_5_-Pyr, 75 Hz): δ 173.41, 171.98, 171.83, (each *C*O), 81.74 (*C*-1), 78.35 (*C*-5), 76.19 (*C*-3), 71.90 (*C*-2), 70.42 (*C*-4), 62.37 (*C*-6), 51.13 (*C*-α), 39.89 (NH*C*H_2_C_13_H_27_), 39.05 (*C*-β), 36.46, 32.08, 32.00, 29.94, 29.88, 29.67. 29.64, 29.61, 29.57, 29.48, 27.25, 26.02, 22.89, 22.85 (each *C*H_2_), 14.22 (overlap of COC_8_H_16_*C*H_3, _NHC_13_H_26_*C*H_3_); HRMS (MS-TOF): [M+Na]^+^ calcd for C_34_H_65_O_8_N_3_Na: calcd: 666.4664 found 666.4689.

*N^4^-β-D-Galactopyranosyl-N^2^-tetracosanoyl-L-asparagine tetradecylamide *(**4**). Triethylamine (0.05 mL) was added to a stirring solution of **19** (0.035 g, 0.035mmol) dissolved in CH_2_Cl_2_/MeOH/H_2_O (1 mL/2 mL/1 mL) at 40 °C. It was stirred for 18 h. The precipitate formed was filtered through a vacuum to afford **4** as a white solid (23 mg, 79%). ^1^H-NMR (300 MHz, *d*_5_-Pyr): δ 10.20–10.14 (m, 1 H, N*H*COCH_2_), 9.02–8.90 (m, 1 H, N*H*COC_23_H_47_), 8.50 (t, *J *= 5.5 Hz, 1 H, CON*H*C_14_CH_29_), 5.90 (dd, *J* = 8.9 Hz, *J *= 17.9 Hz, 1 H, *H*-1), 5.65–5.61 (m, 1 H, *H*-α), 4.73 (d, *J* = 6.1 Hz, 1 H, *H*-4), 4.60 (d, *J* = 2.9 Hz, 1 H, *H*-2), 4.56–4.36 (m, 3 H, overlap of *H*-6, *H*-6', *H*-3), 4.20–4.13 (m, 1 H, *H*-5), 3.49–3.38 (m, 2 H, NHC*H*_2_C_13_H_27_), 3.29–3.21 (m, 2 H, *H*-β, *H*-β'), 2.35 (t, *J* = 7.5 Hz, 2 H, COC*H*_2_C_22_H_45_), 1.77–1.73 (m, 2 H, COCH_2_C*H*_2_C_21_H_43_), 1.59–1.54 (m, 2 H, NHCH_2_C*H*_2_C_12_H_25_), 1.26 (bs,62 H, overlap of COC_2_H_4_(C*H*_2_)_20_CH_3_, NHC_2_H_4_(C*H*_2_)_11_CH_3_), 0.89–0.85 (m, 6 H, overlap of COC_8_H_16_C*H*_3_, NHC_13_H_26_C*H*_3_); HRMS (MALDI MS-QTOF): [M+Na]^+^ calcd for C_48_H_93_O_8_N_3_Na: calcd: 862.6860 found 862.6841.

## 4. Conclusions

In summary, we present a short and convenient route to access glycolipid mimetics from suitably protected and commercially available L-aspartic acid building blocks and easily synthesized galactosyl amines. A small collection of compounds of diverse structural characteristics has been prepared. The design of suitably assembled building blocks and careful consideration of the synthetic sequence, to avoid undesired side reactions, will allow for the next generation of glycolipid mimetics bearing different mono or oligosaccharides, as well as fatty acid derivatives of different chain lengths and saturation patterns.
